# Change in the Faculty

**Published:** 1853-12

**Authors:** 


Change in the Faculty.—The former incumbent of the Chair of Theory and Practice of Medicine in the Iowa Medical Department, has been prevented from filling his usual position during the present course of Lectures, in consequence of personal duties and engagements.

The vacancy thus made has been ably filled by the appointment of Freeman Knowles, M. D., a medical gentleman of large experience and a thorough familiarity with the principles and practice of medicine. Dr. K. has spent some years in distant parts of the continent; has had a wide observation of disease in various countries and climates, and has already entered upon his duties with energy and success.

